# Identification of a major QTL underlying sugar content in peanut kernels based on the RIL mapping population

**DOI:** 10.3389/fpls.2024.1423586

**Published:** 2024-07-04

**Authors:** Feifei Wang, Huarong Miao, Shengzhong Zhang, Xiaohui Hu, Chunjuan Li, Ye Chu, Charles Chen, Wen Zhong, Tianyu Zhang, Heng Wang, Linying Xu, Weiqiang Yang, Jing Chen

**Affiliations:** ^1^ Shandong Peanut Research Institute, Qingdao, China; ^2^ Department of Horticulture, University of Georgia, Tifton, GA, United States; ^3^ Department of Crop, Soil, and Environmental Sciences, Auburn University, Auburn, AL, United States; ^4^ Shandong Seed Administration Station, Jinan, Shandong, China; ^5^ Rizhao Agricultural Technical Service Center, Rizhao, Shandong, China; ^6^ Cixi Agricultural Science Research Institute, Cixi, Ningbo, Zhejiang, China

**Keywords:** peanut, whole genome re-sequence, sucrose, soluble sugar, QTL

## Abstract

High sugar content in peanut seeds is one of the major breeding objectives for peanut flavor improvement. In order to explore the genetic control of sugar accumulation in peanut kernels, we constructed a recombinant inbred line population of 256 F_2:6-7_ lines derived from the Luhua11 × 06B16 cross. A high-resolution genetic map was constructed with 3692 bin markers through whole genome re-sequencing. The total map distance was 981.65 cM and the average bin marker distance was 0.27cM. A major stable QTL region (*qSCB09*/*qSSCB09*) was identified on linkage group (LG) B09 associated with both sucrose content (SC) and soluble sugar content (SSC) explaining 21.51-33.58% phenotypic variations. This major QTL region was consistently detected in three environments and mapped within a physical interval of 1.56 Mb on chromosome B09, and six candidate genes were identified. These results provide valuable information for further map-based cloning of favorable allele for sugar content in peanut.

## 1 Introduction

Peanuts (*Arachis hypogaea* L.) are widely cultivated worldwide as food and oil crops. Peanuts are not only used as a rich source for vegetable oil extraction, and are also rich in protein and micronutrients (Toomer, 2018). In recent years, the increase in both production and consumption of fresh roasted peanuts has resulted in demands for improved sweetness in peanut kernels, which is highly correlated with flavor (Pattee et al., 1998; Guo et al., 2023b). The content of soluble sugars in the kernels will directly determine peanut sweetness (Guo et al., 2023b). The color and flavor of roasted peanut depends on peanut sugar content and its interaction with other components under heating conditions (McDaniel et al., 2012). Soluble sugars in peanut include sucrose, fructose, and glucose, with sucrose being for the highest proportion (McDaniel et al., 2012).

Sucrose, a key product of photosynthesis, serves as a primary long-distance translocated carbon for sink metabolism in higher plants (John Farrar and Gallagher, 2000; Li et al., 2021). Numerous candidate genes related to sucrose metabolism and accumulation, such as those sucrose synthase (SUS), sucrose phosphate synthase (SPS), sucrose phosphate phosphatase (SPP), invertase (INV), seed expressed water-soluble sugar transport protein (SWEET) family, and sucrose transporter (SUT) genes, have been reported in soybean and other species (Chen et al., 2015; Zhen et al., 2018; Wang et al., 2019). Additionally, raffinose synthase (RS), protein kinase (PK) and several transcription factors (TFs), including WRKY, MYB and bZIP (basic leucine zipper) have been implicated in regulating sucrose accumulation (Dierking and Bilyeu, 2008; Kumar et al., 2010; Wei et al., 2018; Chen et al., 2020; Li et al., 2020; Yu et al., 2021). In peanut, the transcriptome analysis identified 28 DEGs involved in sucrose metabolism, including the genes encoding seed expressed water-soluble sugar transport protein (SWEETs), cell wall invertase (CWIN), vacuolar invertase (VIN), cytoplasmic invertase (CIN), Sucrose synthase (SUS), hexokinase (HK), and sucrose-phosphate phosphatse (SPP) (Li et al., 2021).

Genetic analysis suggested that sucrose content in peanut kernels is controlled by two additive–dominance–epistasis major genes plus an additive–dominance–epistasis polygene (Qin et al., 2021). However, a lack of favorable alleles or genes has hindered breeding progress, given that the sugar content of seeds is influenced by various factors, including abiotic and biotic stresses, environmental conditions such as temperature, soil moisture, freezing, seed maturity, growth conditions, and genotype (Maughan et al., 2000; Pattee et al., 2000; Knizia et al., 2023; Guo et al., 2023b). Quantitative trait locus (QTL) mapping is a powerful method to identify alleles or genes controlling complex traits. Over the past few decades, increasing number of peanut QTLs associated with sucrose content have been reported by BSA-seq, GWAS, and genetic mapping (Li et al., 2023; Guo et al., 2023a, 2023b; Zhang et al., 2023b; Huai et al., 2024; Wang et al., 2024). Although these studies made advances in dissecting the genetic mechanism of sucrose content, these is still much to be learned about this trait in peanut. In particular, it is still difficult to identify the most promising QTL for genetic cloning.

In this study, a population of 256 recombinant inbred lines (RILs) was utilized to map the QTLs for sucrose content and soluble sugar content in peanut kernel via high throughput sequencing. The objectives of this study were: (1) to construct a high-density genetic map (HDGM) according to the whole genome re-sequencing protocol; (2) to determine genomic regions that are associated with the sucrose content and soluble sugar content for kernels; (3) to identify the candidate genes for the major stable QTLs.

## 2 Materials and methods

### 2.1 Plant material and phenotyping

An F_2:6_ populations of 256 RIL lines derived from a cross between ‘Luhua11’ and ‘06B16’ was developed at the experimental station of Shandong Peanut Research Institute, Qingdao, China. ‘Luhua11’ cultivar is large-seeded with low sugar content. The genotype ‘06B16’ is small-seeded with high sugar content. The RIL population and its parental lines were planted in experimental fields in Laixi (N 36.86°, E 120.53°), Shandong Province (planted in May and harvested in September of 2021 and 2022); in Greenhouse of Laixi (N 36.86°, E 120.53°), Shandong Province (planted in May and harvested in September of 2022); and Weihai (N 37.24°, E 122.37°), Shandong Province (planted in May and harvested in September of 2023). The field and greenhouse experiments both followed a randomized block design with three replicates. For each plot, 10 plants from each RIL line were grown 15-cm apart within a row, with an 85-cm gap between RILs. Standard agricultural practices were applied for field management. Each plant was harvested individually at its maturity to prevent loss from over-ripening. Only eight plants in the middle of each row were used for trait measurement. About 20 seeds from each line were used to calculate seed sugar concentration using near-infrared reflectance (NIR) spectroscopy following the manufacturer’s protocol (Spectra Star XL, Unity, USA) (Tang et al., 2018).

### 2.2 Statistical analysis of phenotypic data

The mean value and standard deviation of each trait for the parents and each RIL line were analyzed, and Student’s *t*-tests were conducted using IBM^®^ SPSS^®^ statistics 19. The normality of the population data was analyzed using Kolmogorov-Smirnov tests. Using the equation *h* ^2^=*σ*_g_^2^/(*σ*_g_^2^+*σ*_ge_^2^/n+*σ*_ϵ_^2^/nr), the broad-sense of heritability(*h* ^2^) for traits was calculated using ANOVA analysis with QTL IciMapping V4.2 (Meng et al., 2015).

### 2.3 DNA extraction, resequencing and SNP calling

At the seedling stage, young leaf tissue from recombinant inbred lines (RIL) lines and both parental plants were collected for DNA isolation. Genomic DNA was sheared to 350 bp fragments using a Covaris^®^ disruptor. Subsequently, the DNA fragments underwent a series of steps in the library preparation process, including end repair, addition of polyA tails, addition of sequencing adapters, purification, and PCR amplification, culminating in the completion of the entire library preparation process (Illumina Inc., San Diego, CA, USA). High-throughput sequencing was performed on an Illumina Nova6000 platform at Lianchuan Biotechnology Corporation (Hangzhou, China), to generate 150-bp paired-end reads. The sequencing depths for the two parents and the RILs were approximately 20× and 3×, respectively. After filtering the reads to eliminate adapters and low-quality reads, the remaining clean reads were aligned to the reference genome (https://data.legumeinfo.org/Arachis/hypogaea/genomes/Tifrunner. gnm2.J5K5/) (Bertioli et al., 2019) using the BWA-0.7.10. The uniquely mapped reads were used to call SNPs with SAMtools v0.1.19 and GATK v3.3.0. Homozygous and polymorphic SNPs between the two parents were selectively retained for analysis within the RIL population. SNPs meeting the criteria of a missing rate and heterozygosity rate both less than 10%, along with a sequencing depth exceeding 3×, were employed for subsequent analyses and the construction of the genetic map.

### 2.4 High density genetic map construction

SNPs were consolidated through binning using the genotype information of the parents. SNPBinner software (Gonda et al., 2019), which employs a Hidden Markov model (HMM) to determine recombination breakpoints and construct co-separation bins, was utilized in this study. To build a high-quality linkage map, bins with over 50% of samples with missing genotypes and severe partial separation were discarded. QTL IciMapping V4.2 software was applied to construct the linkage map, employing the 2-opt algorithm and performing rippling local optimization with a window size of 5 (Meng et al., 2015). The genetic positions of markers on each linkage group (LG) are presented in **Supplementary Table 4**. In addition, a co-linearity map was generated to evaluate the map quality. The linkage group number corresponds to the chromosome number assigned by the Tifrunner reference genome, indicating a high degree of consistency between genetic and physical maps.

### 2.5 QTL mapping

The R/qtl package (Broman et al., 2003) was used to detect QTL and confirm the relationship between different markers around each QTL with the composite interval mapping method (CIM). The permutation test was conducted 1000 times with probability larger than 1% as the cutoff value. A logarithm of the odds (LOD) threshold value of 2.5 was employed to declare the presence of a QTL at a significance level of 99%. The positive and negative additive effect that represented the favorable alleles were from ‘Lu11’and ‘06B16’, respectively.

### 2.6 Candidate gene mining and expression analysis

All the genes in the QTL region were annotated in four databases (NR, Swiss-Prot, GO, KEGG). Transcription data of genes in different tissues were obtained from the PeanutBase and used to graph a heatmap at https://software.broadinstitute.org/morpheus/ (Clevenger et al., 2016). A heatmap indicating the relative expression levels of candidate genes was constructed at https://www.omicstudio.cn/tool. Secondary structure prediction for candidate protein was performed with an online tool (PSIPRED) at http://bioinf.cs.ucl.ac.uk/psipred.

## 3 Results

### 3.1 Phenotypic variations of sugar content in parents and RIL individuals

**Figure 1:**
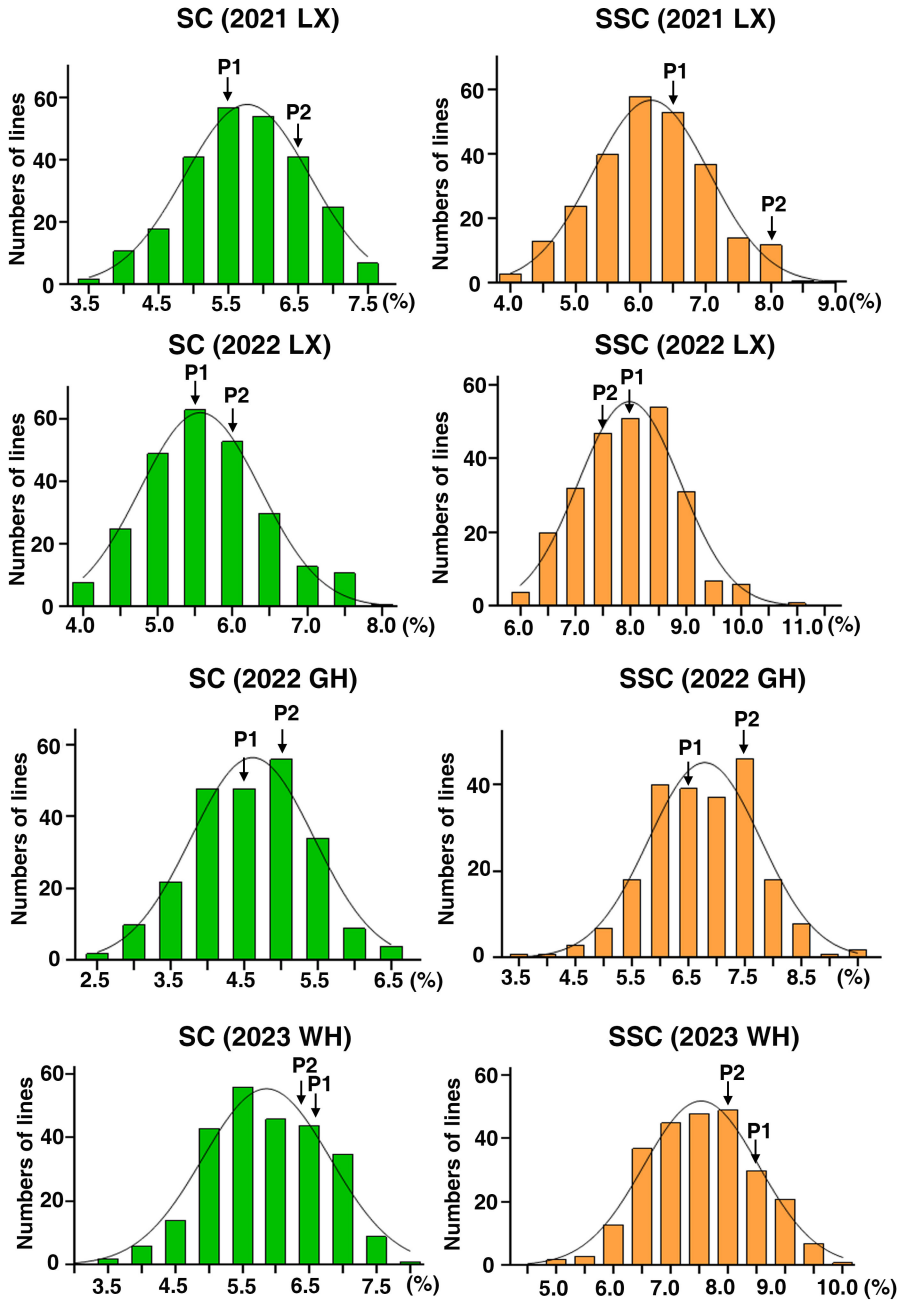
Phenotypic distribution of SC and SSC in the RIL population across four environments. The y-axis represents the number of lines and the x-axis represents the values of sugar content (SC) and Soluble sugar content (SSC). 2021LX, 2022LX, 2022GH, and 2023WH were four trials in Laixi (LX), Weihai (WH) and Greenhouse (GH) from 2021 to 2023. P1 and P2 denote the parents ‘Luhua 11’ and ‘06B16’, respectively.

The mature peanut kernels of ‘06B16’ had higher sucrose and soluble sugar content (5.91 ± 0.61% and 7.75 ± 0.32% on average, respectively) than those of ‘Lu11’ (5.49 ± 1.00% and 7.47 ± 0.95%, respectively) across four environments (**Table 1**). Phenotypic data of the RIL population demonstrated skewed distribution (**Figure 1**; **Table 1**), indicating polygenic inheritance of these two traits. Transgressive segregation was observed in most environments (**Figure 1**). Sucrose content in the RIL population ranged from 3.33% to 7.55% in the 2021 Laixi, 3.81% to 8.08% in the 2022 Laixi, 2.35% to 6.54% in the 2022 Greenhouse, 3.46% to 7.88% in 2023 Weihai. The soluble sugar content of the RIL population ranged from 3.80% to 8.76% in the 2021 Laixi, 6.08% to 10.98% in the 2022 Laixi, 3.60% to 9.35% in the 2022 Greenhouse, 4.98% to 9.82% in the 2023 Weihai. ANOVA (analysis of variance) results indicated that the effects of genotype (G), environments (E), and interaction of G and E (G ×E) were all significant (**Table 2**). The sucrose content and soluble sugar content exhibited relatively high broad-sense heritability (*h^2^*), ranging from 0.78 to 0.71 (**Table 2**).

**Table 1:** Phenotypic variation of Soluble sugar content and Sucrose content among the RIL population of four environments.

Trait	Environment	Parents		RIL population					
		Luhua11	06B16	Mean ± SD^a^	Min^b^	Max^c^	Skew	Kurt	Sig. of K-S test^d^
SC(%)	2021 LX	5.82	6.30	5.74 ± 0.81	3.33	7.55	-0.168	-0.329	0.200
	2022 LX	5.22	5.94**	5.65 ± 0.82	3.81	8.08	0.351	-0.103	0.200
	2022 GH	4.27	5.03	4.57 ± 0.05	2.35	6.54	-0.112	-0.381	0.000**
	2023 WH	6.64	6.35	5.84 ± 0.86	3.46	7.88	-0.086	-0.532	0.200
	Mean **±** SD	5.49 ± 1.00	5.91 ± 0.61						
SSC(%)	2021 LX	6.89	8.05	6.16 ± 0.90	3.80	8.76	0.040	-0.011	0.200
	2022 LX	7.99	7.54	7.95 ± 0.87	6.08	10.98	0.203	-0.018	0.200
	2022 GH	6.46	7.41	6.75 ± 0.06	3.60	9.35	-0.184	0.123	0.000**
	2023 WH	8.52	7.99	7.53 ± 0.94	4.98	9.82	0.007	-0.422	0.200
	Mean **±** SD	7.47 ± 0.95	7.75 ± 0.32						

a SD, standard deviation.

b Min, minimum value.

c Max, maximum value.

d Sig of K-S test, significance for normality test by Kolmogorov-smirnov.

**means significant p<0.01.

2021LX, 2022LX, 2022DP, and2023WH were four trials in Laixi (LX), Greenhouse (GH) and Weihai (WH) from 2021 to 2023.

**Table 2:** Analysis of the broad-sense of heritability of sucrose content and soluble sugar content.

Traits	Source	DF^a^	SS^b^	MS^c^	*F* value	*P*	*h^2^*
SC	G	255	695.19	2.73	17.97	<0.01	0.78
	E	3	407.05	135.69	894.27	<0.01	
	G×E	740	524.37	0.71	4.67	<0.01	
	Error	896	135.95	0.15			
SSC	G	255	717.62	2.81	10.02	<0.01	0.71
	E	3	906.32	302.11	1075.80	<0.01	
	G×E	740	775.85	1.05	3.73	<0.01	
	Error	896	251.62	0.28			

a *DF*, degree of freedom.

b *SS*, sum of square.

c *MS*, mean of square.

### 3.2 Resequencing and SNP identification

Approximately 2362.75 G cleaned data (Q20 > 94%) were generated, including 15.75 billion reads (**Supplementary Table 1**). About 368.86 and 304.57 million reads were obtained for the female and male parents, respectively, whereas 40.58–200.37 million reads were obtained for the RILs (**Supplementary Table 1**). More than 99% of the reads were mapped to the reference genome (**Supplementary Table 2**). A total of 924,882 polymorphic SNPs were identified for the two parents, of which 286,582 were homologous and used to screen for SNPs in the RILs. GATK software was also used to detect variation, the uniformly output SNPs were retained as reliable and used for the following analysis (**Supplementary Table 3**).

### 3.3 Construction of the high-density genetic map

**Figure 2:**
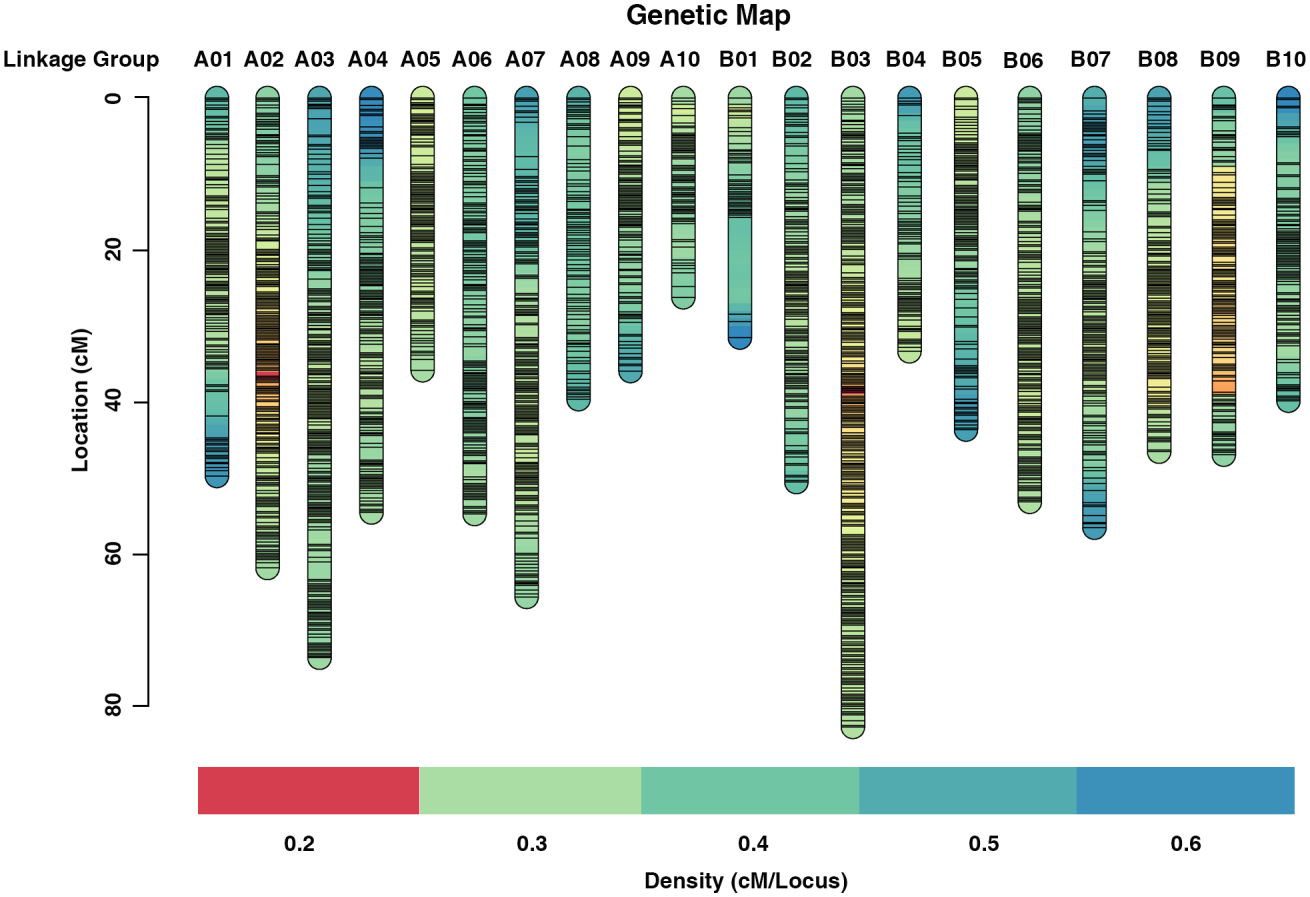
High density genetic map of the RIL population using Bin markers, the markers were indicated by black bars.

Redundant markers were removed from the filtered SNP set using SNPBinner (Gonda et al., 2019). In total, 3,875 bin markers (83,642 SNPs) were detected. After filtering the low quality bin markers, 3692 bin markers (82,292 SNPs) were used to construct the high density genetic map (HDGM) and were assigned to 20 linkage groups (LGs) (**Figure 2**; **Supplementary Table 4**). The whole map length was 981.65cM, with the genetic distance ranging from 26.18 cM to 82.74 cM for each LG with an average marker distance of 0.27cM (**Figure 2**; **Table 3**). LG B03 was the longest group covering a distance of 82.74 cM with 401 loci, while LG A10 was the shortest group spanning 26.18 cM with 91 loci (**Table 3**). Subsequently, the degree of map uniformity and inter-marker linkage were evaluated by the percentage of ‘Gaps ≤ 5 cM’, which ranged from 98.80% to 100% with an average value of 99.94%. The largest gap existed on LG B01 which was 12.77 cM. In order to evaluate the precision of the high density genetic map, we performed collinearity analysis by comparing the genetic positions of markers on each LG with their corresponding physical positions. The collinearity between the genetic and genomic positions was remarkably high, with all values surpassing 99.99% (**Supplementary Table 5**; **Supplementary Figure 1**), providing strong evidence of a thoroughly organized marker assignment.

**Table 3:** Summary of the high-density genetic map.

LG^a^	Marker number	Genetic distance (cM)	Average distance (cM)	Maximum gap (cM)	Gaps <5 cM (%)
A01	175	49.73	0.29	3.14	100
A02	293	61.77	0.21	1.49	100
A03	264	73.58	0.28	2.33	100
A04	185	54.47	0.3	3.97	100
A05	159	35.8	0.23	1.43	100
A06	181	54.74	0.3	1.93	100
A07	231	65.58	0.29	4.54	100
A08	115	39.55	0.35	1.75	100
A09	128	35.91	0.28	1.63	100
A10	91	26.18	0.29	2.11	100
B01	84	31.52	0.38	12.77	98.80
B02	177	50.48	0.29	1.57	100
B03	401	82.74	0.21	1.1	100
B04	112	33.28	0.3	2.53	100
B05	147	43.62	0.3	1.92	100
B06	221	53.08	0.24	1.27	100
B07	188	56.46	0.3	2.84	100
B08	191	46.49	0.24	2.5	100
B09	228	46.86	0.21	1.58	100
B20	121	39.81	0.33	3.46	100
Total	3692	981.65	0.27	12.77	–

### 3.4 Quantitative trait loci identification for sugar content

**Figure 3:**
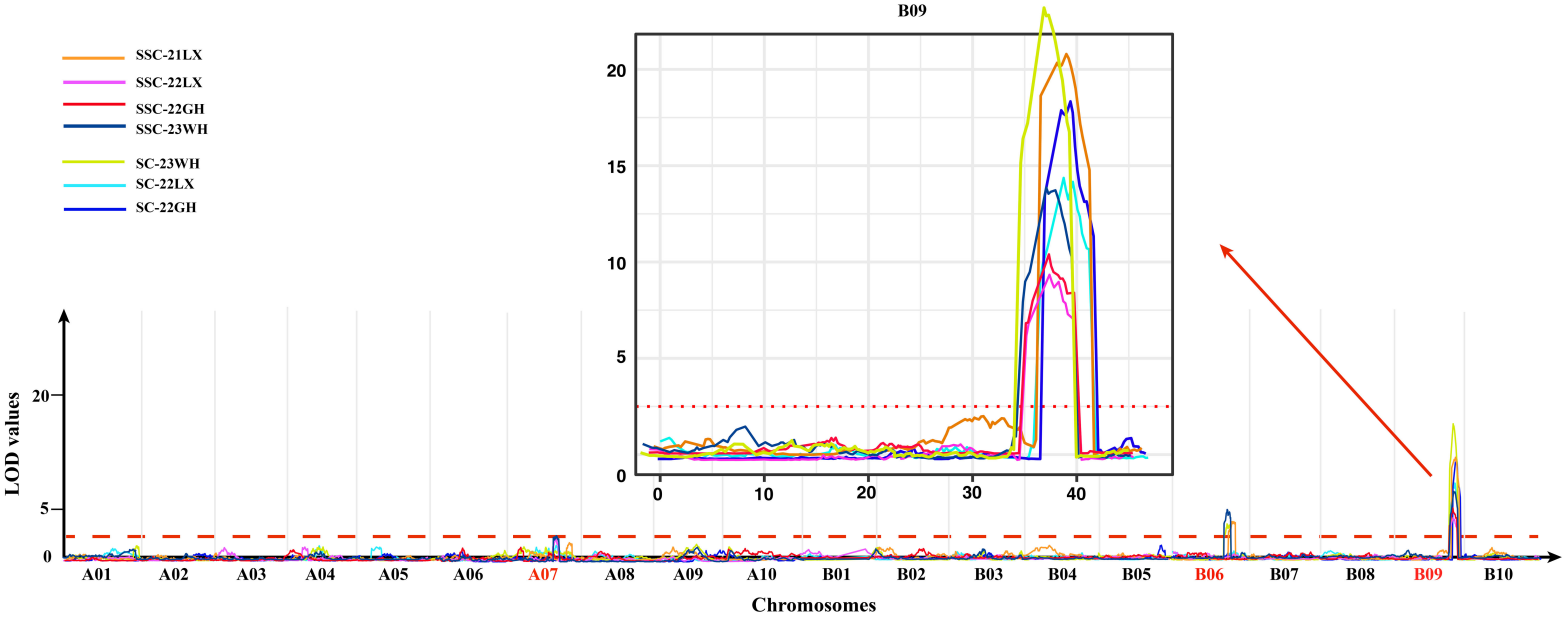
LOD curves of SC and SSC on the whole genome across four environments. The chromosomes of QTLs identified were denoted in red, and the major QTL region in chromosome B09 was showed in details.

A total of five QTLs for sucrose content with 4.61-33.58% phenotypic variation explained (PVE) were mapped on 3 chromosomes including LG A07, B06, and B09 based on the HDGM with LOD values of 2.59-22.75 (**Figure 3**; **Table 4**). Among them, a major QTL region on LG B09 (*qSCB09.1*, and *qSCB09.2*) was stably expressed across three environments with 21.51%-33.58% PVE. Donor alleles for *qSCB09.1*, and *qSCB09.2* were from ‘06B16’. The remaining two QTLs were identified in single environment.

**Table 4:** QTL analysis for sucrose content and soluble sugar content.

Trait	Env^a^	QTL	LG^b^	CI^c^	Flanking Markers	Physical position (Mb)	LOD^d^	PVE^e^	ADD^f^ (%)
SC	22LX	*qSCA07*	A07	40.79-41.20	C07P46749447- C07P48513103	45.61-48.64	2.59	4.61	0.17
	23WH	*qSCB06*	B06	38.00-42.90	C16P136994717- C16P140732082	136.84-140.76	6.02	10.27	0.22
	22GH	*qSCB09.1*	B09	37.16-41.85	C19P154679799- C19P155780993	154.20-155.80	16.77	28.20	-0.40
	22LX	*qSCB09.2*	B09	36.50-41.18	C19P154108125- C19P155713851	154.06-155.76	13.30	21.51	-0.37
	23WH		B09	36.50-41.18	C19P154108125- C19P155713851	154.06-155.76	22.75	33.58	-0.47
SSC	22LX	*qSSCA07.1a*	A07	39.96-42.43	C07P41451767- C07P53315323	41.13-53.33	3.28	5.79	0.20
	22LX	*qSSCA07.2a*	A07	43.06-43.06	C07P53492321- C07P53492321	53.45-53.54	2.65	4.70	0.18
	21LX	*qSSCA07.1b*	A07	52.27-52.47	C07P76632324- C07P77230657	76.11-77.31	2.65	4.66	0.18
	21LX	*qSSCA07.2b*	A07	53.52-53.52	C07P77571599- C07P77571599	77.51-77.63	2.51	4.42	0.17
	23WH	*qSSCA07c*	A07	39.55-44.31	C07P34356686- C07P63228123	32.75-63.80	3.38	5.90	0.19
	21LX	*qSSCB06a*	B06	39.63-44.53	C16P138656520- C16P141175869	138.53-141.26	7.54	12.69	0.25
	23WH	*qSSCB06b*	B06	38.20-42.90	C16P137246479- C16P140732082	137.15-140.76	9.25	15.32	0.31
	22GH	*qSSCB09.1*	B09	36.50-41.18	C19P154108125- C19P155713851	154.06-155.76	10.02	18.85	-0.39
	22LX		B09	36.50-41.18	C19P154108125- C19P155713851	154.06-155.76	9.68	16.15	-0.34
	23WH		B09	36.50-41.18	C19P154108125- C19P155713851	154.06-155.76	12.70	20.43	-0.39
	21LX	*qSSCB09.2*	B09	37.16-41.85	C19P154679799- C19P155780993	154.20-155.80	20.19	30.45	-0.46

a *Env*, environment.

b *LG*, linkage group.

c *Cl*, confidence interval.

d *LOD*, logarithm of the odds.

e *ADD*, additive effect.

f *PVE*, phenotypic variation explained.

QTL mapping identified 11 QTLs for soluble sugar content with 4.42%-30.45% PVE. These were distributed on LG07, B06, and B09 (**Figure 3**; **Table 4**). The major QTL region was on LG19 (*qSSCB09.1*, and *qSSCB09.2*) was consistently detected at all four environments, with LOD scores of 9.68–20.19 (**Table 4**). The additive effect was between -0.46 and -0.34, implying the high soluble sugar content allele was derived from the male parent ‘06B16’. The other seven QTLs were detected in single environment.

**Figure 4:**
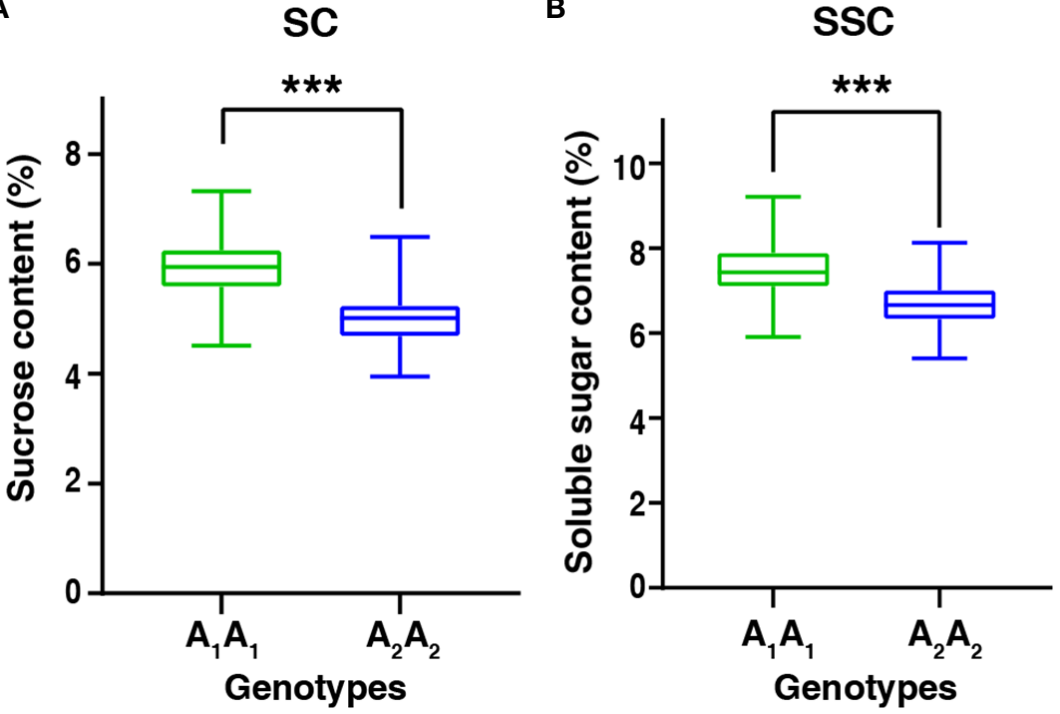
The boxplot of sucrose content and soluble sugar content between two genotypic groups in the RIL population. A_1_A_1_ and A_2_A_2_ represented the elite allele in *qSCB09* and *qSSCB09* were from 06B16 and Lu11, respectively. ***represent significant difference at *P*=0.001 level by *t*-test.

**Figure 5:**
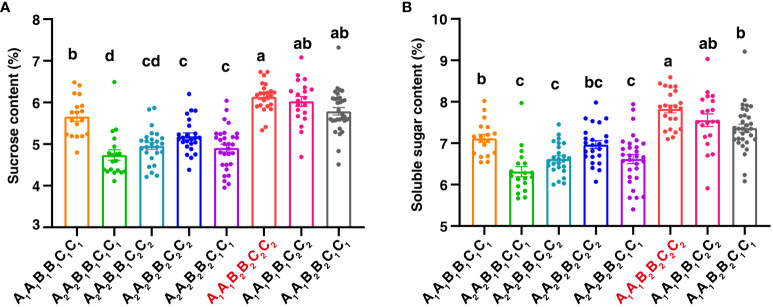
The combinatorial effects of QTLs on B09, A07, and B06 with sucrose content **(A)** and soluble sugar content **(B)**. The letters A, B, C represented QTL regions on B09, A07, and B06, respectively. A_1_A_1_ and A_2_A_2_ represented the homozygous alleles in *qSCB09* and *qSSCB09* were from 06B16 and Lu11, respectively. Different letters indicate significant (p < 0.05) differences between means, based on a one-way ANOVA test.

It is interesting that *qSCB09* and *qSSCB09* were co-localized to a 4.02 cM interval (37.16–41.18 cM, the corresponding physical interval is 154.20-155.76 Mb) with flanking markers of C19P154679799- C19P155713851 (**Table 4**). To determine phenotypic contributions of this QTL region, flanking marker profile was used to selected and group RIL into two homozygous genotypes (**Figure 4**; **Supplementary Table 6**). Student *t*-test revealed significant differences (P < 0.01) between the two genotypic groups (**Figure 4**). The RILs with the A_1_A_1_ genotype (representing the ‘06B16’ allele in *qSCB09* and *qSSCB09*) had a higher sucrose content and soluble sugar content than those with aa genotype (representing the ‘Lu11’ allele in *qSCB09* and *qSSCB09*) (**Figure 4**). The elite allele of *qSSC/SCA07* and *qSSC/SCB06* from the elite ‘06B16’ was defined as the ‘B_1_B_1_’ and ‘C_1_C_1_’ genotype, respectively, while another allele from ‘Lu11’ was defined as the ‘B_2_B_2_’ and ‘C_2_C_2_’ genotype, respectively. We noticed that some RILs exhibited higher sucrose and soluble sugar contents compared to both parents. Therefore, we further examined the distribution of allele from all three QTLs, *qSSC/SCB09*, *qSSC/SCA07* and *qSSC/SCB06*. The genotype of three QTLs was ‘A_1_A_1_B_1_B_1_C_1_C_1_’ for parent ‘06B16’ and ‘A_2_A_2_B_2_B_2_C_2_C_2_’ for parent ‘Lu11’. There were eight different genotypes, ‘A_1_A_1_B_1_B_1_C_1_C_1_’, ‘A_1_A_1_B_1_B_1_C_2_C_2_’, ‘A_1_A_1_B_2_B_2_C_2_C_2_’, ‘A_2_A_2_B_1_B_1_C_2_C_2_’, ‘A_2_A_2_B_2_B_2_C_2_C_2_’, ‘A_2_A_2_B_1_B_1_C_1_C_1_’, ‘A_2_A_2_B_2_B_2_C_1_C_1_’, and ‘A_1_A_1_B_2_B_2_C_1_C_1_’, from the lines in the RIL population. Obviously, the lines with ‘A_1_A_1_B_2_B_2_C_2_C_2_’ genotype by combining three elite alleles of *qSSC/SCB09*, *qSSC/SCA07* and *qSSC/SCB06* showed an over-dominant phenotype with 6.19% of sucrose content, and 7.89% of soluble sugar content, which is significantly higher than that of lines with other genotypes (**Figure 5**; **Supplementary Table 6**). These results showed that the combination of three elite alleles from three QTLs, *qSSC/SCB09*, *qSSC/SCA07* and *qSSC/SCB06*, produced an over-dominant phenotype with significantly increased sucrose content and soluble sugar content.

### 3.5 Potential candidate genes within the major QTL *qSCB09/qSSCB09* for sucrose and soluble sugar content

The stable major QTL region *qSCB09/qSSCB09* was mapped in the cultivated peanut genome between 154,057,090 and 155,797,391 bp on chromosome B09 with 118 annotated genes located in this interval. There were 84 functional genes related to maltose metabolism, signal transduction, response to stress, lipid metabolism, secondary metabolite biosynthesis, oxidation-reduction, transcription, sucrose metabolism, and starch biosynthesis (**Supplementary Table 7**).

**Figure 6:**
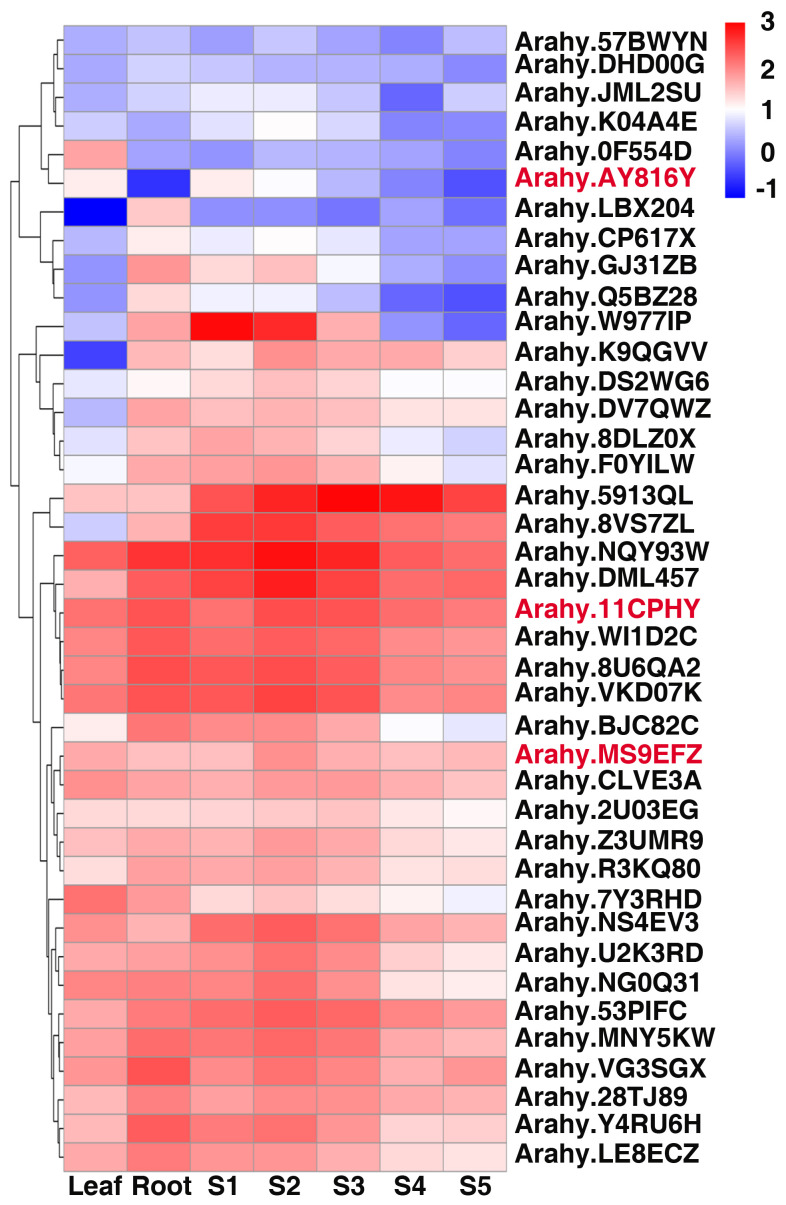
Heatmap analysis of candidate genes of peanut SSC and SC in different developmental stages. S1 to S5 indicated the fruit Pattee 1 to 5 stages. The values of transcript abundances were taken from Clevenger et al. (2016).

To investigate expression pattern of annotated genes in the stable major QTL region *qSCB09/qSSCB09*, we surveyed the transcript abundance of these genes in leaf, root, and seed at different developmental stages. Interestingly, most of the genes in the confidence interval were highly expressed in seeds (**Figure 6**). The expression pattern of *Arahy.MS9EFZ*, *Arahy.11CPHY*, and *Arahy.AY8I6Y* were all first increased and then decreased, which is in line with the trend of sucrose accumulation, indicating they may be candidate genes for sucrose content and soluble sugar content in peanut kernels.

Meanwhile, a total of 38 SNP variants were identified in the interval based on the whole genome resequencing data in the parental lines (**Supplementary Table 8**). Among these variants, seven SNP variants which affected six genes were detected (**Supplementary Table 8**), including two intron variants in *Arahy.JI97RZ* and *Arahy.KA4332*, a premature start codon gain variant in the 5’-UTR of *Arahy.MS9EFZ*, three downstream gene variants in *Arahy.11CPHY*, *Arahy.LC7X94*, and *Arahy.AY8I6Y*, and a missense variant in *Arahy.AY8I6Y*. This SNP (G/C) caused an amino acid substitution (Ser toThr) of Arahy.AY8I6Y and might result in secondary structure (strand, helix and coil) variations (**Supplementary Figure 2**).

## 4 Discussion

In this study, we successfully identified a novel major and stable QTL region, along with candidate genes associated with sugar content in peanut seeds. The QTL region *qSCB09*/*qSSCB09* is the major and stable locus controlling sugar content, explaining phenotypic variance of 21.51% to 33.58%.

The parental lines did not significantly differ for sucrose content and soluble sugar content in this study, but showed transgressive segregation, indicated that much of the broad-sense heritability was contributed by dominant and epistatic gene actions, which can be exploited via hybrid breeding (Ayalew et al., 2022). This phenomenon is often encountered in studies of complex traits in crops, where multiple genetic factors contribute to variations in quantitative traits (Ren et al., 2020; Mackay et al., 2021; Ayalew et al., 2022; Zhang et al., 2023c). It has implications for crop improvement, as it introduces genetic diversity that may lead to enhanced traits. Notably, the absence of extreme phenotypes in the parent lines underscores the dynamic nature of genetic interactions. In this study, the board-sense heritability *(h^2^*) of sucrose and soluble sugars was 78% and 71%, respectively (**Table 2**), The higher broad-sense heritability (92% and 86%) of sucrose content in peanut have been reported (Li et al., 2023; Wang et al., 2024), indicating a potential for sucrose and soluble sugars improvement. However, the concentration of sucrose and soluble sugars were also influenced by environmental factors, such as soil, water, climate, etc. The minimum content of sucrose and soluble sugars in the RIL population varied greatly across different environments, the content of SC and SSC were 2.35%-3.81% and 3.60%-6.08%, respectively, and the highest sucrose and soluble sugars concentrations were found in 22LX (**Table 1**).

Construction of a high density genetic map (HDGM) is a traditional way of mapping QTL for agronomic traits. The whole genome re-sequence (WGRS) strategy has advantages in HDGM and favorable QTL identification (Kumar et al., 2020; Zhao et al., 2020; An et al., 2021; Wu et al., 2023). Currently, several HDGMs based on WGRS have been published in cultivated peanut, and resulted in yield and oil quality related QTLs detection in cultivated peanut (Li et al., 2019; Liu et al., 2020; Wang et al., 2020; Sun et al., 2022; Li et al., 2023; Yang et al., 2023). In our present study, a high-density genetic map was constructed with 3692 bin markers, the whole length was about 981.65 cM and the average marker distance was 0.27cM (**Figure 2**; **Table 3**), the density of which was higher than the previous studies (Li et al., 2023; Wang et al., 2024). Collinearity analysis validated the high quality of this map (**Supplementary Table 5**). In view of the HDGM and the phenotypic data of multiple environments, the QTLs identified in our study were reliable.

There were increasing numbers of quantitative trait loci (QTLs) associated with sucrose content in peanut have been reported. BSA-seq technology was employed to map four QTLs were located on chromosomes A03 and A06. Furthermore, a stable QTL *qSUCA06* (112.37-112.66 Mb) located on chromosome A06 was finely mapped, accounting for 31.95%-41.05% of the phenotypic variance explained (PVE) (Guo et al., 2023b). Two other genomic regions associated with sucrose content, *qSUCA08a* and *qSUCB06a* on chromosomes A08 and B06 respectively, were identified by QTL-seq, a major QTL, *qSUCA08.2*, explained 5.43%-17.84% of phenotypic variation across five environments (Li et al., 2023). Based on whole genome re-sequencing and construction of a high density genetic map, two major stable QTLs for sucrose content, *qSCA06.2* and *qSCB06.2* were mapped on chromosomes A06 (115.0-116.1 Mb) and B06 (147.9-148.6 Mb) (Lei et al., 2022; Wang et al., 2024). Furthermore, Guo et al. (2023a) mapped 10 QTLs located on A01, A07, A10, B01, B03 and B10, accounting for 4.56%-12.25% of PVE, and *qSUCA07* was detected in three environments (Guo et al., 2023a). A genome-wide association study (GWAS) with principal component analysis identified seven significant QTLs associated with total sugars and 22 significant QTLs associated with sucrose content (Zhang et al., 2023b).

In our study, a total of five sucrose-associated QTLs were detected in this study on four chromosomes (LGs A07, B06 and B09). Furthermore, a novel major stable QTL-*qSCB09* was identified, which explained 21.51-33.58% of the phenotypic variation for sucrose across three environments. We identified a QTL on LG B06, the physical interval was 136.84-140.76 Mb, which is different with *qSUCB06.1* (12.33-20.76 Mb), *qSUCB06.2* (20.60-30.22 Mb), *qSCB06.2* (147.9-148.6 Mb) (Li et al., 2023; Wang et al., 2024). *qSUCA07* was found across three environments which explained 7.05%-12.25% of the phenotypic variation for sucrose (Guo et al., 2023a), which was different to *qSCA07* in our study. Few studies have sought to identify soluble sugar-related QTL in peanut, except a QTL on B06 by BSA-seq which was only a 0.7 Mb (149.47-152.28 Mb) physical interval (Zhang et al., 2020), and is close with *qSSCB06* in our study (137.15-141.26 Mb) (**Table 4**). The *qSSCB06* in our study was stable detected in two environments, explaining 12.69%-15.32% PVE (**Table 4**). Interestingly, our results showed that *qSCB09* and *qSSCB09* were co-localized to a 4.02 cM interval (37.16-41.18 cM), indicating a potential simultaneous impact on the contents of sucrose and soluble sugar. Sucrose is the main component of soluble sugar in peanut (Li et al., 2023), thus the main point in breeding high sweetness peanut have been focused on increasing sucrose content. The *qSSCB06* identified in our study suggested that it is possible to increase other soluble sugars (such as fructose and glucose) content in the future. Moreover, this study reveals that the sucrose content and soluble sugar content are controlled by three QTLs or genes. The highest sucrose content and soluble sugar content phenotype can only be achieved when the elite allelic genotype of the three genes is combined.

Several candidate genes related to sucrose content in peanut have been reported*. Arahy. I1MI1E*, a DEG in *qSUCA08.2*, encoding receptor-like protein kinase, was more likely candidate gene responsible for sucrose content. It may control sucrose content via regulating these genes related to sucrose metabolism and transport (Li et al., 2023). *Arahy.Y2LWD9*, which encodes acyl-CoA-binding domain 3 (ACBD), is a candidate gene in *qSUCA06* for sucrose content accumulation (Guo et al., 2023b). *Arahy.42CAD1* was identified as the most likely candidate gene in qSucA06, being co-expressed with genes involved in vesicle transport and oil body assembly. This indicates that sucrose accumulation may be due to disruptions in TAG transport or storage mechanisms (Huai et al., 2024).

Protein kinases were proven to play key roles in sugar accumulation (Lu et al., 2007; Lecourieux et al., 2010; Huang et al., 2014; Li et al., 2023). The candidate gene *Arahy.11CPHY* encoded serine/threonine-protein kinase PRP4, the expression of which increased in S2 and S3 stages and decreased in S4 and S5 stages (**Figure 6**), in accordance with the sucrose accumulation pattern (Li et al., 2021). Notably, *Ah.W977IP* and *Ah.NG0Q3I* which have higher expression in early developmental stages of peanut seed (**Figure 6**), also matched with sucrose accumulation pattern (Li et al., 2021). *Ah.W977IP* and *Ah.NG0Q3I* both encoded alpha-xylosidase 1 which is a Glycosyl Hydrolase 31 (GH31) family member and related to starch and sucrose metabolism (Cuebas-Irizarry et al., 2017; Xu et al., 2023). Thus, they may also be the candidate genes for sucrose content and soluble content in peanut kernels. In our study, *Arahy.AY8I6Y* was most likely the candidate gene for *qSC/SSCB09*. *Arahy.AY8I6Y* encodes a Zinc finger protein CONSTANS-LIKE 16 (COL16), which belongs to zinc finger protein transcription factor family. *COL* genes have been reported to be involved in many molecular regulation processes and plant growth development, including regulation of flowering time, photoperiodic responses and photomorphogenesis, stigma color and key roles in banana (*Musa nana*) fruit ripening (Hassidim et al., 2009; Chen et al., 2012; Ordoñez-Herrera et al., 2018; Zhang et al., 2023a). *COL* genes also involved in abiotic stress tolerance response, anthocyanin accumulation and plants carotenoid metabolism regulation (Bai et al., 2019; Zhang et al., 2023a). Besides, a zinc finger protein AdDof3 has been reported to interacted physically with the AdBAM3L promoter, regulating starch degradation in Kiwiruit (Zhang et al., 2018), indicating a potential role in sugar metabolism. *Arahy.AY8I6Y* was identified as the most likely candidate gene due to a nonsynonymous SNP causing amino acid substitution (Ser to Thr). The predicted structural change associated with missense variation may affect its molecular and biological functions, however, further study is needed to verify the function of *Arahy.AY8I6Y* and its role of regulating sugar accumulation.

Besides, the correlation analysis of sugar content with protein content and oil content were investigated, the result showed that sucrose content and soluble sugar content were all significantly negatively correlated with oil content, but were no correlation with protein content (**Supplementary Table 9**), which is consistent with the reports in soybean (Jiang et al., 2018; Huai et al., 2024). So, we can hypothesis that some genes associated with transformation between sugar and oil may also affect sugar content in peanut.

## 5 Conclusion

In summary, we identified a new major stable QTL region *qSCB09/qSSCB09* for both sucrose content and soluble sugar content of peanut kernels based on whole genome resequencing and QTL mapping. Within this confidence interval, six non-synonymous mutation genes were identified as candidate genes, and *Arahy.AY8I6Y* was the most likely candidate gene for sucrose accumulation in peanut kernel. These findings will contribute to an enhanced understanding of sugar accumulation in peanut kernels, and the candidate genes will be useful for breeding of high sugar content peanut varieties.

## Data availability statement

The original contributions presented in the study are included in the article/**Supplementary Material**. Further inquiries can be directed to the corresponding author.

## Author contributions

FW: Funding acquisition, Investigation, Software, Supervision, Writing – original draft, Writing – review & editing. HM: Formal analysis, Investigation, Writing – review & editing. SZ: Formal analysis, Investigation, Software, Writing – review & editing. XH: Project administration, Resources, Supervision, Writing – review & editing. CL: Data curation, Formal analysis, Writing – review & editing. YC: Conceptualization, Writing – review & editing. CC: Project administration, Writing – review & editing. WZ: Formal analysis, Writing – review & editing. TZ: Data curation, Methodology, Writing – review & editing. HW: Conceptualization, Writing – review & editing. LX: Investigation, Writing – review & editing. WY: Investigation, Validation, Writing – review & editing. JC: Writing – review & editing, Conceptualization, Funding acquisition, Project administration, Resources, Supervision.
