# Supplementary material for: Identification of a major QTL underlying sugar content in peanut kernels based on the RIL mapping population
Source: Front Plant Sci. 2024 Jul 4;15:1423586. doi: 10.3389/fpls.2024.1423586 (PMC11254704; doi:10.3389/fpls.2024.1423586)
Supplement: Supplementary file 1 [file DataSheet_1.docx]

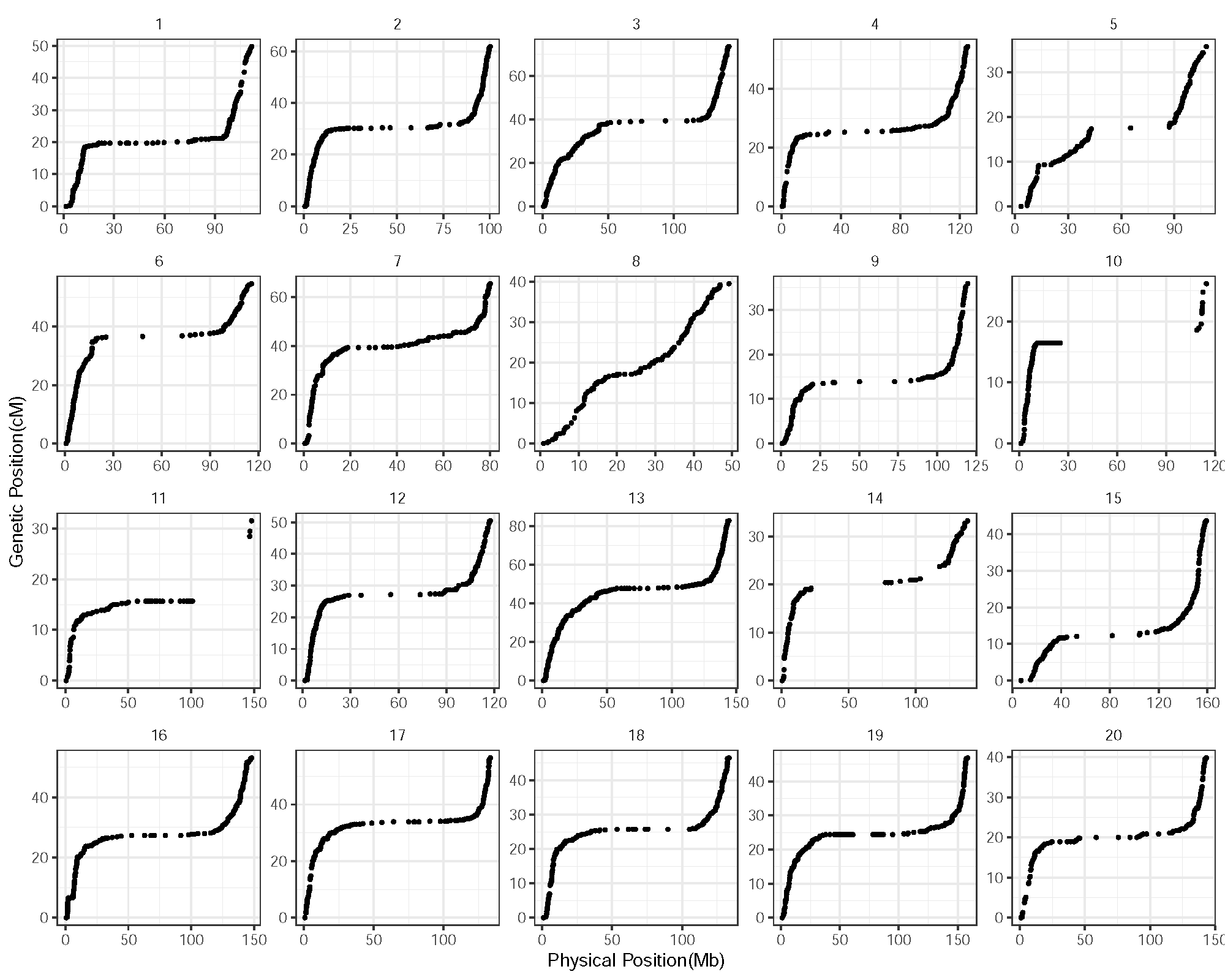


**Figure S1.** Collinearity analysis of the genetic map.


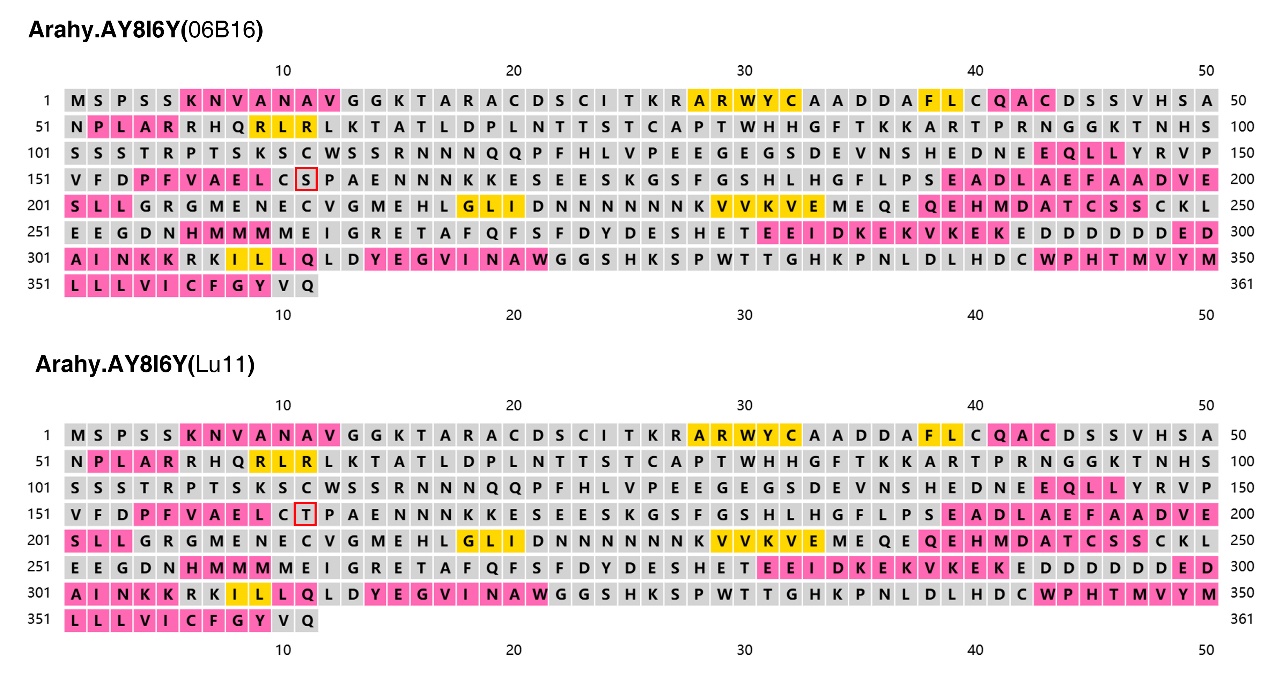


**Figure S2.** Predicted secondary structures of proteins coded by *Arahy.AY8I6Y* alleles. The red frames indicate the amino acid substitution in parents.
